# Substance use patterns and in-hospital care of adolescents and young adults attending music concerts

**DOI:** 10.1186/s13722-017-0105-x

**Published:** 2018-01-09

**Authors:** Stephanie M. Ruest, Alexander M. Stephan, Peter T. Masiakos, Paul D. Biddinger, Carlos A. Camargo, Sigmund Kharasch

**Affiliations:** 1 Section of Pediatric Emergency Medicine, Hasbro Children’s Hospital, Alpert Medical School of Brown University, 593 Eddy St, Claverick 2, Providence, RI 02903 USA; 2 Department of Pediatrics, Massachusetts General Hospital for Children, Harvard Medical School, 55 Fruit St, Boston, MA 02114 USA; 3 Division of Pediatric Surgery, Massachusetts General Hospital for Children, Harvard Medical School, 55 Fruit St, Boston, MA 02114 USA; 4 Department of Emergency Medicine, Massachusetts General Hospital, Harvard Medical School, 55 Fruit St, Boston, MA 02114 USA

**Keywords:** Music concerts, Substance use, Intoxication, Adolescents, Young adults

## Abstract

**Background:**

Few studies describe medical complaints and substance use patterns related to attending music concerts. As such, the objective of this study is to describe patient demographics, substance use and intoxication patterns, and medical interventions provided to adolescents and young adults assessed in an emergency department (ED) for complaints directly related to concert attendance.

**Methods:**

A retrospective chart review of patients 13–30 years old who were transported to the ED directly from music concerts between January 2011 and December 2015 was conducted. Descriptive statistics and logistic regression were used to analyze patient demographic, intervention, and substance use data.

**Results:**

There were 115 concerts identified, of which 48 (42%) were linked to 142 relevant ED visits; the total number of attendees at each concert is unknown. The mean age of the 142 described patients was 19.5 years (SD 3.3) with 72% < 21 and 33% < 18; 71% of patients were female and 96% of visits were substance-use related. Mean blood alcohol level was 242 mg/dL (range 104–412, SD 70). Glasgow Coma Scale (GCS) scores ranged from 3 to 15, with a mean of 14. Two patients required intubation and 61% of patients received interventions, including medications (47%), intravenous fluids (46%), specialty consultation (20%), restraints (14%), imaging (6%), and laceration repair (3%). Attendance at pop and electronic dance music concerts was associated with the widest ranges of GCS scores (8–15 and 6–14 respectively), mass casualty incident declarations, and among the highest mean blood alcohol levels (246 and 244 mg/dL, respectively).

**Conclusions:**

Substance use is the predominant reason for music concert related ED visits and patients may have serious levels of intoxication, receiving multiple medical interventions. These data demonstrate the need for additional large-scale studies to confirm trends and increase awareness of this important public health problem.

## Background

Mass gathering events, such as sporting and political events, music concert events, visits by dignitaries, and others, have been defined as the presence of > 1000 people gathered at a specific location for a defined period of time [1]. Prior research has identified several attributes of mass gatherings that result in morbidity and mortality, including weather and environmental factors [2–6], alcohol and drug use [5–7], crowd attendance and density [8–10], and event type [6, 9, 10]. Music concerts are one unique type of mass gathering event that can occur in indoor or outdoor venues, are often associated with substance use, and are attended by individuals ranging in age from young children to the elderly depending upon the performer. As such, the morbidity and mortality associated with these events may be quite varied. While previous publications on mass gatherings have helped to advance our understanding of these event characteristics [9, 11, 12], only a handful of studies have uniquely addressed complaints directly related to music concert attendance, the majority of which have been cross-sectional event reports rather than longitudinal series, and are from outside of the United States [5–7, 13–18]. This may be because there is currently no universally standardized approach to data collection or large patient registries for reporting adverse outcomes at concerts or other mass gathering events [19].

Despite the limited number of publications on issues related to substance use patterns, levels of intoxication, and the need for medical care at concerts, in recent years there has been frequent media attention describing deaths and significant morbidity related to substance use at music concerts. Additionally, there have been multiple formal declarations of “mass casualty incidents” (MCIs) across the country when emergency medical services (EMS) and medical providers have been faced with large surges of patients, often adolescents and young adults, with significant levels of intoxication, dehydration, injuries, and other issues arising secondary to substance use at both indoor and outdoor concerts [20–30]. Previous publications have reported that adolescents and young adults below the age of 30 are more likely to present for medical care at concerts than older individuals [5–7, 18, 19]. Of note, a 2015 publication cited at least 68 concert-associated deaths that were reported by the press over 15 years [16]. In 2013–2014 alone, journalists reported at least 10 deaths of individuals under the age of 25 and > 650 hospital transports related to drug and alcohol use at concerts in cities throughout the United States, the vast majority of which occurred at electronic dance music (EDM) concerts or festivals [21, 26–29].

Although there is growing interest in these issues, there are no publications known to these authors that document in-hospital care, including patient demographics, substance use patterns, levels of intoxication, and toxicology results, provided to adolescents and young adults who attended shows across multiple music genres over a multi-year study period. The objective of this study was to identify adolescents and young adults evaluated in a large urban emergency department (ED) for complaints directly related to concert attendance over the course of 5 years and to describe these factors listed above based on the music genre of the concert attended.

## Methods

### Design, setting, and population

A retrospective chart review was performed on the identified case series of patients between the ages of 13 and 30 years old seen in the Massachusetts General Hospital (MGH) ED on nights coinciding with concerts at a large nearby event venue between January 2011 and December 2015. This age range was chosen as prior publications have found that concert-goers below the age of 30 are more likely to present for medical evaluation [5, 6, 18]. MGH is a large urban academic medical center located within Boston, Massachusetts, approximately 0.75 miles from the concert venue of interest. The study was reviewed and approved by the MGH institutional review board.

The studied concert venue has a maximum patron capacity of over 19,000 people. Although the venue has on-site EMS, it does not have an on-site physician to evaluate and treat concert patrons. The total number of concert attendees and the number of patients treated and released at on-site first aid stations at each event are unknown to the authors. In part based on its geographic location, MGH is the primary hospital for transport of patients from this concert venue. All patients requiring medical evaluation, with the exception of those rerouted in MCI declarations based on EMS protocol, were directly transported from the venue to MGH (personal EMS communication, December 12, 2014). During the course of the study period, there were two MCI’s resulting in the transport of approximately 30 patients to other area hospitals, although the exact number is unknown to the authors.

Concert dates were identified via publically available event schedules for the venue of interest and the performer and music genre for each concert were recorded. Music genre was designated based on the performer’s official website or the Billboard™ music charts (e.g. pop, rock, electronic dance music, jazz, etc.). Patients seen on the day of the concert through 2 a.m. the following morning who had a chief complaint or discharge diagnosis related to ingestion, intoxication, alcohol or drug use, altered mental status, agitation, loss of consciousness, syncope, near syncope, headache, dizziness, dehydration, chest pain, shortness of breath, injury, trauma, wound, abrasion, or laceration underwent further chart review. Patient stated chief complaints and final ICD-9 primary and secondary discharge codes associated with the visits were provided by MGH health information services. This list of general complaints and diagnoses was chosen based on previous literature describing the most common presentations identified at music concerts [5, 7, 19]. The time frame up until 2 a.m. the following morning was chosen in an attempt to capture all concert-related patients, as concerts generally ended by 11 p.m. Patients were included in the study if they met the above criteria and their medical record included specific documented reference to concert attendance at the venue of interest. Patients were excluded if there was no mention of attending a concert at this venue or if it was not clearly specified in the available documentation.

### Data collection and analysis

Age, sex, time of presentation, verbal report of drug or alcohol use, Glasgow Coma Scale (GCS) score by a triage nurse or first-contact physician on arrival and the lowest documented GCS score during the ED visit, medical intervention(s) (e.g. airway support, imaging, intravenous fluids, laceration repair, medications, restraints, etc.), serum and urine toxicology results, length of stay, final diagnosis, and disposition were abstracted by two physicians (AS and SR). AS performed the primary review of all charts (n = 698) and SR independently reviewed 25% of charts; 95% of re-reviewed charts yielded identical data. SR and AS discussed any discrepancies and came to a consensus for the final data analysis.

The study was mainly descriptive in nature. Data were transcribed into Microsoft Excel 2010 (Microsoft Corporation, Redmond, WA) and then exported into STATA 14.0 (StataCorp LP, College Station, TX) for analysis. Continuous variables were summarized using mean (standard deviation, SD) while categorical variables were summarized using frequency and percentage. Multivariable logistic regression analysis was performed to obtain odds ratios and 95% confidence intervals (CI). Odds ratios were adjusted for age and sex, consistent with a prior publication that controlled for the same covariates [5].

## Results

Between January 2011 and December 2015, 115 concerts were identified via review of publically available schedules. Based on date and time of presentation, age, and chief complaint or discharge diagnoses, 698 patients met inclusion criteria for chart review. After more detailed chart review, 142 patients were eligible for data analysis. Five hundred and forty five patients’ charts were reviewed and excluded because there was no specific reference to concert attendance, and an additional 11 patients were excluded because they came from concerts at venues that were not the main venue of interest.

**Fig. 1:**
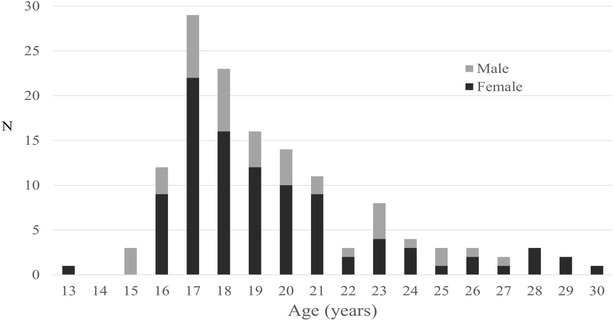
Age and sex distribution of concert attendees seen in the emergency department. Mean age of all patients was 19.5 years (SD 3.3 years), with 72% less than age 21 and 33% less than age 18

Patients ranged in age from 13 to 30 years old with a mean age of 19.5 years (SD 3.3); 72% (n = 102) of patients were under the age of 21 and 33% (n = 47) were under age 18 (Fig. 1). When controlling for sex, individuals who attended rock concerts had 2.4 times the odds (95% CI 1.1–5.3, P = 0.027) of being aged 21 or older as compared with those who attended concerts of all other genres. There were no other statistically significant relationships found between age and the genre of the concert attended. Overall, 71% (n = 101) of patients were female. When controlling for age, individuals who attended pop concerts had 3.5 times the odds (95% CI 1.33–9.17, P = 0.01) of being female as compared with those who attended concerts of all other genres. There were no other statistically significant relationships found between sex and the genre of the concert attended.

**Fig. 2:**
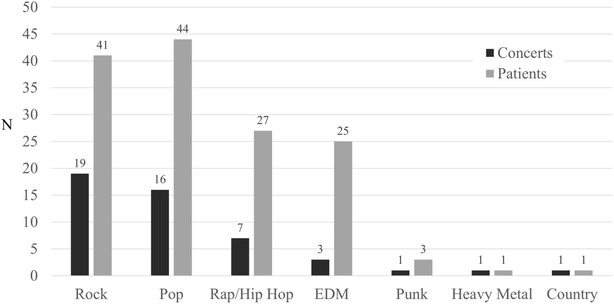
Number of concerts resulting in ED visits compared to the number of patients, by music genre

Forty-eight (42%) of 115 concerts accounted for all identified ED concert-related patient visits (Table 1). Seven music genres were represented among these 48 concerts: country (n = 1), EDM (n = 3), heavy metal (n = 1), pop (n = 16), punk (n = 1), rap/hip hop (n = 7), and rock (n = 19). Overall, 31% of patients attended pop concerts, 29% rock, 19% rap/hip hop, 18% EDM, 2% punk, 1% country and 1% heavy metal (Fig. 2). There were no patients from jazz concerts.

**Table 1:** Concerts that resulted in ED visits

Genre^a^	Performer
Country	Lady Antebellum
Electronic dance music	Avicii^b,c^, Pretty Lights
Heavy metal	Avenged Sevenfold
Pop	Beyonce^c,d^, Britney Spears, Jingle Ball^c,e^, Justin Beiber^c^, Justin Timberlake, Lady Gaga, Madonna, Marc Anthony (Latin Pop), Miley Cyrus^b^, New Kids on the Block, Rhianna^c,d^, Stevie Wonder^d^
Punk	Dropkick Murphys
Rap/hip hop	Drake, Jay-Z, Kanye West, Mackelmore and Ryan Lewis, Monster Jam^c,g^, The Throne Tour with Kanye West and Jay-Z
Rock	Avett Brothers, Black Keys, Bon Jovi, Boston Strong^f^, Coldplay, Dave Matthews, Dispatch^h^, Foo Fighters, Imagine Dragons, Kings of Leon, Mumford and Sons, Red Hot Chili Peppers, Tool, U2^h^, Van Halen

^a^As per the artist’s official website or Billboard™ music charts

^b^Concert resulted in an emergency medical services mass casualty incident (MCI) declaration

^c^Two separate concerts by the same performer resulted in ED visits

^d^Beyoncé, Rhianna, and Stevie Wonder are identified as both pop and rhythm and blues (R&B). For the purposes of this study, they were categorized as pop

^e^Multiple performer show, mostly pop performers

^f^Multiple performer show, mostly rock performers

^g^Multiple performer show, mostly rap/hip hop performers

^h^Three separate concerts by the same performer resulted in ED visits

Two specific concerts in the study period, one pop and one EDM, resulted in large volumes of patients requiring EMS transport, leading to a formal declaration of an MCI and distribution of patients to MGH and other local area hospitals according to the established Boston EMS MCI plan. The total number of patients transported from the pop concert is unknown to these authors, however, per press reports of the EDM concert, there were 50 patients who were treated and released on scene and an additional 36 patients transported to area hospitals, 13 of whom were seen at MGH [23].

Overall, 96% (n = 137) of all patient presentations involved alcohol or drug use based on patient report and/or clinician documentation. While the majority of patients did present with a chief complaint related to substance use, one patient with the complaint of chest pain, one with pre-syncope, one with migraine symptoms, two with agitation and three with injuries all were discharged with a primary or secondary diagnosis related to substance use. The five presentations that did not involve a primary or secondary diagnosis of intoxication or substance use included two complaints of chest pain, two lacerations, and one complaint of dizziness/pre-syncope.

**Fig. 3:**
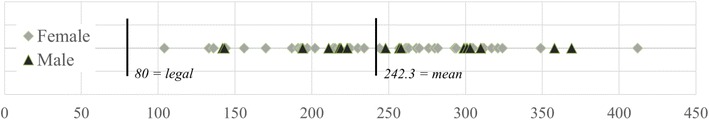
Serum blood alcohol levels (mg/dL) in tested patients. N = 60. Mean blood alcohol level (BAL) for all patients was 242.3 mg/dL (range 104.3–412.0). Mean BAL for females was 247.5 mg/dL (104.3–412.0) and mean BAL for males was 243.8 mg/dL (142.2–358.0)

All 142 patients were asked about alcohol use. Of those, 87% (n = 124) of patients reported using alcohol on the day of the ED visit. Of the 38 individuals who were specifically asked about whether or not they had consumed alcohol prior to entering the concert venue, 71% (n = 27) answered affirmatively, 21% (n = 8) denied this, and 8% (n = 3) refused to answer the question. Forty-three percent of individuals (n = 60) had serum toxicology screening sent at the discretion of the evaluating clinician to assist with medical decision-making and/or management and 98% of those screens were positive for alcohol, with an overall mean blood alcohol level (BAL) of 242 mg/dL (range 104–412, SD 70), which is equivalent to a blood alcohol content of 0.24% (SD 0.07%) (Fig. 3). Punk, pop and EDM shows had the highest mean BAL’s of 311 mg/dL (SD 0), 246 mg/dL (SD 92) and 244 mg/dL (SD 48), respectively. Ten individuals who denied alcohol use had serum toxicology screens sent; all were positive with a mean BAL of 262 mg/dL (SD 36). Conversely, an additional 6 patients who denied alcohol use did not have screens sent; two were described as clinically intoxicated, two had lacerations, one each had dizziness, chest pain, and multiple injuries due to an assault.

Overall, 123 out of 142 individuals had documentation that they were asked about drug use. Of those, 18% (n = 22) reported drug use, 81% (n = 100) denied drug use, and < 1% (n = 1) refused to answer the question. Only 20 individuals had urine toxicology screening sent at the discretion of the clinician; screens were not necessarily sent on individuals who endorsed drug use. Of those sent, 50% were positive for one or more drugs, including opioids, marijuana, and amphetamines. Of note, one of the tested patients reported chronic opiate use for pain, one patient reported recreational ecstasy use, one reported prescribed methylphenidate use, and one reported recreational methylphenidate use.

The Glasgow Coma Scale (GCS) is a neurological scale used by medical providers to assess a patient’s level of consciousness by evaluating an individual’s eye, verbal, and motor responses to various stimuli. A low score of 3 denotes deep unconsciousness or coma whereas a high score of 15 denotes a fully awake and alert individual. The GCS score may be used to help guide medical decision making, such as determining the need for tracheal intubation in the setting of a low or precipitously declining score. In this study, scores were obtained via nursing or physician documentation. The mean GCS score on arrival for all patients was 14 (SD 1.8, range 6–15), and the mean lowest documented GCS for all patients was 13 (SD 2.5, range 3–15). Patients from EDM and pop concerts demonstrated the widest range of GCS scores on arrival, with means of 13 (SD 3, range 6–15) and 14 (SD 2, range 8–15), respectively. The lowest documented GCS score for all patients was 3, which was a subsequent GCS score recorded in a patient who attended an EDM show and who required intubation. Although 80% of patients did not have a difference between their arrival GCS and their lowest documented GCS score, 10% dropped 1–2 points, 5% dropped 3–4 points, and nearly 5% dropped ≥ 5 points. Of those with a decrease of ≥ 5 points, four were female and one was male, with ages ranging from 17 to 24 years, initial GCS scores of 12–15, and BAL’s ranging 194–310 mg/dL.

While some patients only required clinical monitoring, 61% (n = 86) received interventions beyond clinical observation (Table 2). Two individuals required intubation for airway protection (1%), 1 had a nasopharyngeal airway placed (1%), 67 received medications (47%), 65 received intravenous fluids (46%), 19 required either physical restraints, chemical restraints, or both due to significant agitation (13%), 8 had imaging performed (6%), and 4 had laceration repairs (3%). There were 29 individuals (20%) who had specialty consultations, which included social work, plastic surgery, psychiatry, and pediatric intensive care.

**Table 2:** Interventions, length of stay, and disposition of individuals seen in the ED (total patient n = 142)

	N (%)
Was any intervention received beyond clinical observation^a^	
Yes	86 (61)
Airway support	
Intubation	2 (1)
Nasopharyngeal airway	1 (< 1)
Imaging	8 (6)
Intravenous fluids	65 (46)
Laceration repair	4 (3)
Medications	
Antiemetics	49 (35)
Other^b^	30 (21)
Restraints	
Physical	17 (12)
Chemical	14 (10)
Both	12 (9)
Specialty consultations	
Any consult	29 (20)
Social work	22 (16)
Pediatric ICU	2 (1)
Plastic surgery	2 (1)
Psychiatry	3 (2)

	Mean (SD)
Overall mean length of stay (h)	
Min–Max 0.4–18.6	4.3 (3)
Mean ED length of stay, by musical genre	
Country (n = 1)	3.7 (–)
EDM (n = 25)	5.1 (3.0)
Heavy metal (n = 1)	2.9 (–)
Pop (n = 44)	4.0 (3.4)
Punk (n = 3)	6.2 (4.0)
Rap/hip hop (n = 27)	5.4 (4.7)
Rock (n = 41)	3.4 (2.2)

	N (%)
Patient disposition	
Treated and released	128 (90)
Left without being seen/without completing treatment	11 (8)
Admitted	2 (1)
Discharged to another hospital	1 (< 1)

^a^Interventions: airway support (nasopharyngeal airway or intubation), imaging, intravenous fluid administration, laceration repair, medication administration, restraints (chemical, physical, or both), and specialty consultation

^b^Other medications given: pain medications, antibiotics, anxiolytics, rapid sequence intubation medications, and electrolyte repletion

The mean length of stay across all music genres was 4.3 h (SD 3.4), with a range of 0.4–18.6 h. The shortest mean length of stay was for individuals attending heavy metal concerts (2.9 h, SD 0), and the longest mean length of stay was for those who attended punk concerts (6.2 h, SD 4). Overall, 90% of patients were treated and released with an admission rate of less than 2%. The remainder of patients left without being seen or left without completing treatment.

## Discussion

This study examined a series of 115 music concerts across multiple genres and described the presentations, demographics, substance use data, and interventions provided to individuals transported from a large music venue to an urban medical center for evaluation of complaints related to concert attendance. While there were no mortalities, 96% of visits involved substance use, the majority of patients were females under the age of 21, and > 60% of patients were provided with some type of intervention and/or treatment by medical providers.

Although prior studies have broadly evaluated the public health implications of mass gatherings, most of the concert-related literature to date has described the on-site care of single specific concerts or festivals and is based on non-U.S. populations. The lay-press reports of at least 10 deaths and over 650 hospital transports related to drug and alcohol use at concerts throughout the U.S. in 2013–2014 highlight the gap in our understanding of this important public health issue. Previous to this study, Chan et al. is the only study known to the authors that has addressed the in-hospital experience of patients who attended concerts (pop and rock only in their series) over an extended period of time; however, this study included patrons from events other than concerts and only 31% of their patients had alcohol or drug related complaints. Additionally, as their data was from over 15 years ago, it may not reflect the scope of substance use patterns and need for medical care by today’s concert-going youth [18].

Prior studies have reported a sex disparity for patients who present for medical attention, with a published range of 57–69% females comprising the patient population [5, 6, 16, 31]. In this study population, there was also a female predominance, with females comprising 71% of patients. It has been hypothesized that this sex disparity may be related to a disproportionate number of young females attending some of the high patient-volume concerts, an increased likelihood of females becoming ill from substance use, and/or females presenting for care more often [5, 6]. Because the authors do not know the sex breakdown of concert attendees at each of the shows in the current sample, it is not possible to speak further to the possible causes of the female predominance in this study population.

Alcohol and drug use were a common reason for EMS transport from the concert venue to the MGH ED. While prior studies have reported a range of 11–48% of patients presenting from music concerts with substance use-related complaints, 87% of all patients in this study explicitly reported alcohol use and 98% of those tested had a positive serum toxicology screen, with a mean BAL over three times the legal limit for operation of a motor vehicle (80 mg/dL or 0.08%) [7, 18, 31]. Co-ingestions were also present in this series, identified by positive toxicology screens and/or patient verbal reports of tetrahydrocannabinol (THC) and amphetamine use (including methylenedioxy-methamphetamine, otherwise known as MDMA, “molly”, or ecstasy). The proportion of substance-use related presentations in this series was more consistent with that of Lund et al’s more recent population of EDM concert-goers, in which 79% of presentations involved substance use [16]. Over 70% of the patients in this series that were asked about when they consumed alcohol reported that they had used alcohol prior to entering the concert venue. A study by Chapman et al. highlighted the fact that many concertgoers will consume a large portion or all of their alcohol or drug supply before entering a venue to avoid confiscation, which is likely the case among this study population as well [32]. The mean BAL’s were quite high in this patient population, possibly because patrons are often consuming large volumes of alcohol within a short period before entering the venues.

In this series, EDM, pop, rap/hip hop and rock concerts represented the genres with the highest patient volumes. Similar to prior studies, this data supports that rock and EDM are associated with higher rates of presentation, and EDM shows may result in higher acuity of presentations [16, 17, 33]. EDM represented only 6% of concerts in this series, yet resulted in 18% of the patient volume, an MCI declaration, one of the highest mean blood alcohol levels measured, and the lowest mean GCS of patients presenting to the MGH ED. Congruent with the New York City Department of Health and Mental Hygiene’s publication investigating adverse health events from a local EDM festival in 2013, this series demonstrates that some patients who have attended concerts may arrive with significantly altered mental status and in need of close monitoring and more significant medical interventions [34].

The current publication adds a missing piece to the full spectrum of concert-related patient presentations by describing the in-hospital experience at a large Boston teaching hospital over 5 years and across multiple music genres. Lay-press articles and pre-hospital publications have demonstrated that attendance at music concerts may be associated with significant morbidity and mortality and that there is a need for on-site evaluation and treatment of patients by medical providers. This and other publications suggest that patrons attending EDM, pop and rock concerts and patrons who are female and under the age of 21 may be at greatest risk factors for needing medical assessment and care. When patrons present with higher acuity complaints, nearby hospitals must be prepared to care for these patients with significant intoxications and poly-substance ingestions. Medical providers should keep the various types of substances used at these concerts in mind, including timing to peak effects, when creating a treatment and disposition plan for patients, and local hospitals may consider increasing staffing on nights that could have higher patient volumes.

In Canada, harm reduction strategies, including free water, cool down stations, female-only break areas, pill/drug-checking stations, and on-site drug education programs and medical services have all been employed at EDM festivals with success. With these strategies in place at a 2014 seven day EDM festival with over 67,000 cumulative attendees, less than 1% of patrons who presented for care required transport to hospitals, there were no intubations, and no fatalities [17]. The acceptance and implementation of similar health and harm reduction strategies at large U.S. venues for concert-going patrons should be considered by venue owners as a means of decreasing the severity of substance related outcomes. Furthermore, increasing education and awareness for the patrons through public service announcements and on site education tables may also have a positive impact on outcomes. Adopting standardized data collection strategies at venues, including collecting patron demographics, presenting complaints, pertinent exam findings, and disposition plans may increase knowledge of substance use trends and lead to improved preparedness, both at the venue itself and by local EMS providers and hospitals.

## Limitations

This study has some important limitations. The retrospective nature makes it dependent on publicly available information for concert date listings as well as on specific ED documentation of the patients’ concert attendance. All patients without specific reference to concert attendance were excluded, so additional patients who were present at a concert and lacked this documentation may have been missed. Although the authors included a number of medical complaints and diagnoses in the initial medical chart retrieval based on the most common concert-related presentations described in previous publications, an exhaustive search of charts for all patients seen on the days of concerts was not done and, therefore, may have missed additional patients. If true, these data may under-represent the number of patients associated with concert attendance; the extent of the problem may well be greater.

Additionally, while concert date listings are public record and allowed for the targeted search of patients’ hospital records analyzed in this study, both the total number of attendees present at each concert and the number who were evaluated and released by on-site first aid stations are proprietary data that were not made available by the operators of the private concert venue studied despite repeated attempts to obtain this information. Thus, the authors are unable to comment on the relative prevalence of concert-related morbidity between specific music genres. Furthermore, data obtained from the hospital records of those concert attendees who required transport to an ED by EMS is likely skewed towards younger patrons and those with greater levels of intoxication or more serious medical complaints.

While almost all toxicology testing obtained was positive, not all patients were screened; as such, all patients who used alcohol or other drugs prior to or during the concert may not have been identified. However, 87% of patients had laboratory-confirmed or verbal report of alcohol use. It is important to note that individuals who used marijuana in the preceding days to weeks may test positive for THC even if marijuana was not used on the day of the attended concert; nevertheless, the majority of patients either verbally endorsed use that day or showed signs of intoxication at the time of their ED visit, suggesting that positive screens in this study likely represented current use. Also, it should also be mentioned standard urine toxicology panels screen for phencyclidine, barbiturates, cannabinoids, amphetamines, benzodiazepines, opiates and cocaine metabolites, and some drugs, including synthetic marijuana and inhalants, are not detectable on routine toxicology screening, possibly resulting in false-negative screens.

Lastly, this single-venue study does not allow for larger scale predictions of substance use trends and in-hospital interventions and outcomes across all music genres at a variety of indoor and outdoor venues. The venue included was an indoor venue and did not host concerts of all genres. Outdoor country music concerts, for example, have been associated with large patient volumes in the lay press and were not represented in this series [28]. The authors encourage the design and implementation of multi-center, multi-venue studies that include venue-provided data on the total number of concert patrons and data from patrons treated and released by on-site medical providers combined with data on patrons transported to local hospitals.

## Conclusions

The data from this study demonstrate that music concerts can result in the transport of adolescent and young adult patients to emergency departments, many of whom require a high level of medical care and receive significant interventions. In this retrospective case series, the preponderance of female patients, the frequency of alcohol intoxication and other drug use, the considerable number of patients with altered mental status, and the large number of interventions provided to this population, speaks to the substance use behavior among adolescents and young adults attending concerts. Although the sample size of this study limits the ability to predict large-scale trends, specific genres such as pop, EDM, and rock, as shown in this series, may be associated with higher risk as compared with other genres.

The present study provides in-hospital data, including toxicology, GCS, and medical intervention data, that have not previously been reported in the mass casualty medicine literature. It supports the need for additional large-scale collaborative studies as well as universally standardized patient registries to identify patient volume and substance use trends at a national level, along with morbidity and mortality at music concerts. Such efforts may improve venue and local ED planning and preparedness on the days of these events and raise public awareness regarding the scope of adverse outcomes associated with substance use at concerts. Furthermore, the development of collaborative relationships among venues hosting concerts, EMS officials, police, lawmakers, and local hospitals may allow for improved interventions targeted around the well-known relationship between concert attendance and substance use.

## Abbreviations

BAL: blood alcohol level
EDM: electronic dance music
ED: emergency department
EMS: emergency medical services
GCS: Glasgow Coma Scale
MCI: mass casualty incident
MGH: Massachusetts General Hospital
THC: tetrahydrocannabinol
